# Novel Association of the *Presenilin-1* (Leu282Arg) Mutation with Isolated Spastic Paraparesis: Case Presentation and Review of Current Evidence

**DOI:** 10.3390/jcm14176150

**Published:** 2025-08-30

**Authors:** María De la Sen-Domínguez, Manuel Delgado-Alvarado, Marta Torres-Barquin, Remedios Quirce, Javier Riancho

**Affiliations:** 1 Service of Neurology, University Hospital de Valdecilla-IDIVAL, 39011 Santander, Spain; maria.de-la-sen@alumnos.unican.es; 2 Service of Neurology, Hospital General Sierrallana-IDIVAL, 39300 Torrelavega, Spain; manuel.delgado@scsalud.es; 3 Centro de Investigación en Red Enfermedades Neurodegenerativas (CIBERNED), 28029 Madrid, Spain; 4 Service of Radiology, Hospital General Sierrallana-IDIVAL, 39300 Torrelavega, Spain; marta.torres@scsalud.es; 5 Service of Nuclear Medicine, University Hospital de Valdecilla-IDIVAL, 39011 Santander, Spain; mremedios.quirce@scsalud.es; 6 Department of Medicine and Psychiatry, University of Cantabria, 39005 Santander, Spain

**Keywords:** Alzheimer’s disease, amyloid angiopathy, PSEN-1, spastic paraparesis

## Abstract

**Background**: Although *Presenilin-1* (PSEN1) mutations are classically associated with early-onset Alzheimer’s disease (AD), spastic paraparesis (SP) may occasionally represent as an initial or even isolated clinical manifestation. **Methods**: We report the novel association of a PSEN1 mutation (Leu282Arg) with isolated SP at onset in a patient with a family history of early-onset AD. Additionally, we reviewed previously published cases describing similar presentations related to PSEN1 mutations. **Results**: The age of reported patients ranged from 24 to 60 years. The most common clinical course included the presence of cotton wool plaques and a progressive development of cognitive decline following the onset of SP. A positive family history of either motor or cognitive symptoms was consistently observed. **Conclusions**: Our findings emphasize the clinical importance of considering PSEN1 mutations in the differential diagnosis of patients presenting with spastic paraparesis, particularly in the presence of cognitive symptoms, cerebral amyloid angiopathy, or a family history of AD.

## 1. Introduction

Spastic paraparesis (SP) is a motor alteration firstly described in 1940 [1], characterized by mild or moderate loss of motor function accompanied by spasticity in the lower extremities [2]. Its appearance has been associated with different syndromes, including varAD, a variant of Alzheimer’s disease, which presents with SP, dementia, and typically the neuropathological finding of cotton wool plaques (CWPs). This syndrome has been associated with the genetic finding of exon 9 PSEN-1 deletion [3,4].

More than 300 mutations of the Presenilin-1 (PSEN-1) gene have been described. Among them, its association with autosomal-dominant Alzheimer’s disease (ADAD) is one of the most widely known, but it has been associated with an extensive list of diseases [5,6,7], highlighting the need to broaden the knowledge of this gene and its related phenotypes [8,9,10].

In this article, we report a patient with a positive family history of early-onset Alzheimer’s disease (EOAD) and cerebral amyloid angiopathy (CAA) who presented with SP, and we review the cases with PSEN-1 mutations previously published in the literature that presented in the same way.

## 2. Case Report

**Figure 1:**
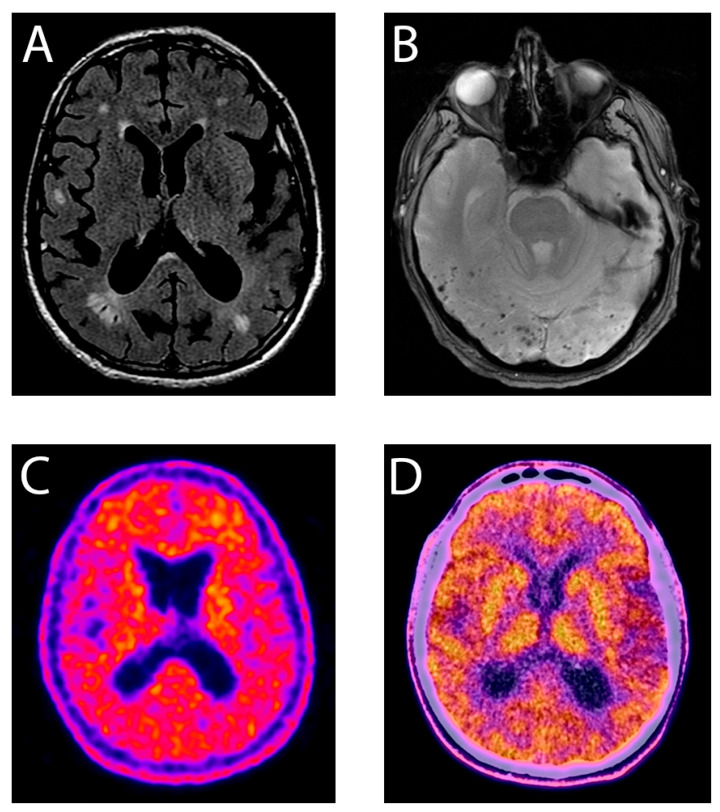
FLAIR (**A**) and echo-gradient (**B**) MRI sections showing hyperintense periventricular and subcortical lesions and multiple lobar microhemorrhages compatible with CAA, respectively. PiB PET and 18-FDG scan demonstrating generalized cortical amyloid deposition (**C**) and subtle decrease in metabolism in the insular area (**D**).

A 54-year-old right-handed patient who had recently moved into our region came to our clinic because of an 8-month course of a clinical picture of loss of strength and progressive clumsiness in gait. Having already been studied in another center, an MRI had been made, showing seemingly demyelinating periventricular lesions (Figure 1). In addition, several biochemical, microbiological, and immunological testing had been carried out, including a spinal fluid study looking for oligoclonal banding, all of which were negative. Systemic and neurological examinations revealed mild paraparesis, with lower-limb hyperreflexia and a spastic paraparetic gait. Taking all these findings into consideration, extended assays were conducted. Nutritional, microbiological, and immunological assessments were all normal (Table 1), while the neurophysiological study showed subtle aberrations in motor evoked potentials of lower limbs.

**Figure 2:**
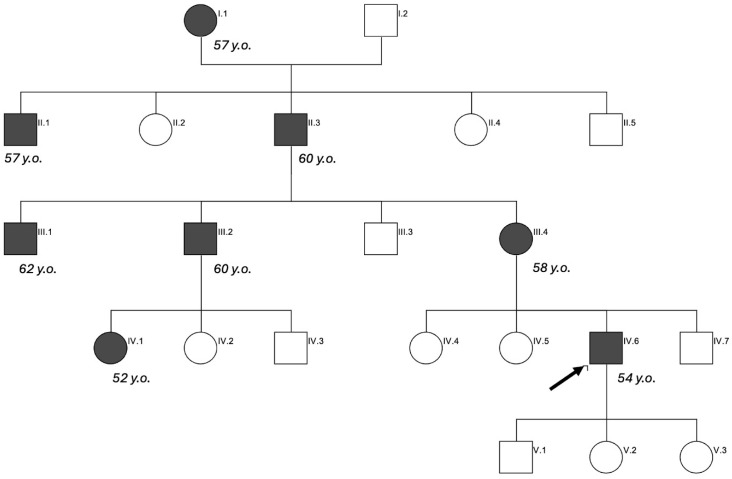
Pedigree diagram illustrating the patient’s family history of presenile AD. Ages at symptoms onset are also represented. Arrow: Index case.

Complementarily, a re-evaluation of the MRI revealed remarkable disturbances in the echo-gradient sequence, consistent with CAA (Figure 1). Importantly, although the patient had no cognitive complaint, he reported having a maternal family history of EOAD. His mother (58 y.o.), cousin (52 y.o.), grandfather (60 y.o.), great uncle (57 y.o.), and several maternal uncles had been diagnosed with presenile AD (Figure 2). An extended genetic panel (Table 1) identified a pathogenic variant in heterozygosis in the PSEN1 gene c.845T>G; p.(Leu282Arg). A later PET- C11 PIB study confirmed amyloid deposition and described a widespread cortical pattern compatible with AD (Figure 1).

Over a two-year follow-up period, the patient exhibited a subtle depressive syndrome accompanied by a progressive deterioration of lower-limb motor function. Despite retaining the ability to ambulate for over 20 min with assistance, the clinical progression necessitated the use of bilateral canes, reflecting a measurable decline in functional mobility. Regarding cognitive function, the patient continues to report no subjective memory complaints or other cognitive difficulties. Nevertheless, a recent neuropsychological evaluation identified subtle deficits, including dyscalculia, executive dysfunction, and mild impairment in free recall memory.

## 3. Discussion

In 2021, 13% of the 226 pathogenic variants that had been reported in PSEN1 were reportedly related to SP [8,9]. Out of them, only around 7% presented, as in our case report, with isolated pure SP at onset [8,9]; its main genetic, clinical, imaging, and neuropathological features are summarized in Table 2.

The PSEN1 gene encodes a core component of the γ-secretase complex and has been increasingly recognized for its role in neurodegeneration. By 2023, the number of pathogenic variants reported had risen from 226 in 2021 to more than 300 [5]. This rapid growth in identified variants underscores the expanding importance of PSEN1 in the study of neurodegenerative diseases. While our review focused on its association with isolated spastic paraparesis (SP) at onset, a broad spectrum of clinical phenotypes has been linked to PSEN1 mutations, ranging from neurodegenerative disorders [5] to dermatological conditions [5]. Beyond its well-established role in autosomal-dominant Alzheimer’s disease, associations with frontotemporal dementia (FTD) [5] and amyotrophic lateral sclerosis (ALS) [6,7] have also been reported.

The precise mechanisms underlying these diverse manifestations remain incompletely understood. Current hypotheses include PSEN1 functioning either as a causative gene or as a locus for genetic modifiers [3]. In addition, both amyloid-dependent and amyloid-independent pathways have been implicated in mediating its phenotypic effects [5], highlighting the complex and multifactorial role of PSEN1 in neurodegenerative disease.

The influence of PSEN1 mutation on the pathogenicity of neurodegenerative diseases has not yet been well understood. Some authors hypothesize about the existence of a threshold effect that, when exceeded, leads to its variable neuropathological and clinical features [3], explaining that the pathogenic route of the disease may be able to run different paths [11]. Among those potential pathways, it is widely believed that its different genetic alterations may play a role in altering the equilibrium between the Beta-amyloid substance (ratio Aβ42/40 [5]), which is in accordance with most of the affected individuals featuring CWP in their neuropathological and imaging analysis [9]. However, this theory may not be suitable, for instance, for the already identified disease varAD, since there are mutations that cause an even greater alteration in this ratio (with higher levels of Aβ42) and do not present spastic paraparesis association [3,12]. Moreover, PSEN-1 mutations usually also result in an elevated ratio of apoptosis by amyloid-independent pathways, such as mitochondrial deficits or calcium imbalance [5].

With respect to the specific mutation detected, Leu282Arg, it was identified previously in a woman who started to show signs of dementia at the age of 49 and was afterwards clinically and histologically diagnosed with AD [13] (1998). However, to the best of our knowledge, this is the first report linking this particular mutation to an initial clinical presentation characterized by isolated SP.

Regarding its clinical characteristics, our case falls within the age range reported in previous studies, with most patients presenting between 24 and 60 years of age. Notably, both our patient and the case described by [14] were initially misdiagnosed as multiple sclerosis, underscoring the importance of considering PSEN1 mutations in the differential diagnosis of SP.

Some of these patients belonged to reported pedigrees of families with EOAD [3,15], and most of the remaining ones had a recorded familial history of motor impairment or dementia, as was the case with our patient. In addition, contrary to our patient, where the clinical appearance of memory failure is still lacking, most patients developed cognitive decline shortly after their diagnosis (see Table 2) [8]. Interestingly, most available reports suggest that individuals with motor impairment as the initial manifestation tend to experience a comparatively better quality of life and/or longer survival [16] than those whose clinical onset is marked by memory decline. This consistent observation strengthens the hypothesis put forward by Brooks et al. [3,9], which posits the presence of a potential genetic modifying locus within the *PSEN1* gene. Such a mechanism could partially account for the clinical heterogeneity observed across pedigrees and may have significant implications for both prognosis and targeted therapeutic strategies.

Finally, in our case report, magnetic resonance imaging did not show CWP as may be expected. In contrast, and curiously, despite still not showing signs of the cognitive decline of an AD profile, CAA was detected. However, it may happen the same way as in the Aus-1 reported pedigree (see Table 1), where different branches of the family exhibit CWP and classic plaques [4].

In conclusion, the detection of PSEN-1 mutations is starting to shed light on the relationship between previously apparent disconnected clinical presentations. It unveils an explanation of the differential evolution and prognosis of formerly seemingly overlapping cases at onset, while it allows its distinction from other phenotypically similar diseases [17,18]. Regardless, its clinical onset as isolated SP still represents a minority of the cases, and understanding its mechanism may potentially have a remarkable impact.

To conclude, due to their potential association with both motor and cognitive syndromes, clinicians should consider PSEN-1 mutations when assessing a patient with either or both former complaints, especially if a family history including any of the clinical diagnoses that may suit the broad spectrum of this mutation’s consequences is to be found.

**Table 2:** Pedigrees and cases reported with clinical onset of spastic paraparesis and mutations in PSEN1.

Author (year)	Age of Onset	PSEN1 Mutation	MRI and Neuropathology	Coexistence of CI During the Course	Familial History
**Kwok et al.** [15] (1997)	45	Δ9 (splice site)	-	++ (Dementia)	-
**Kwok et al.** [15] (1997)	47	Δ9 (G→T exon 9, splice acceptor mutation)	-	++ (Pseudobulbar Palsy)	MI +, CD ++
**Kwok et al.** [15] (1997)	37	R278T (exon 8, missense mutation)	-	++ (Dementia)	-
**Crook, et al**. [19] (1998)	48–64	Δ9 (not splice site) ΔE10	CWP, CAA, DCT, NFTs	++ (Dementia)	MI ++, CD ++
**Sato et al.** [20] (1998)	46	ΔE10 (splice acceptor) AG→AA heterozygous substitution	NFTs, SP	++ (Dementia)	MI ++, CD +
**Houlden et al.** [11] (2000)	20–42	P436Q	CWP	-	-
**Houlden et al.** [11] (2000)	34–38	Del IM p.Ile83Met84del	CWP	-	-
**Smith et al.** [16] (2001)	46–50	ΔE10 (5’9 kb deletion)	CWP, CAA, DCT	PIII:10. +	MI +, CD ++
**Mann et al**. [21] (2001)		ΔE10 (splice acceptor)	CWP ++, CAA, Core Plaques	-	-
**Sodeyama et al**. [22] (2001)	31	Phe237Ile	Diffuse cerebral Cortical Atrophy	+	-
**Jacquemont et al**. [23] (2002)	54	Pro264Leu	Cortical Atrophy +	++	MI +, CD +
**Matsubara-Tsutsui et al**. [24] (2002)	33–45	G266S (exon 8, missense mutation)	Parietal Atrophy	++ (Dementia)	MI ++, CD +
**Tabira et al.** [4] (2002)	32	P284L	Cerebellar Atrophy +++, CWP, NFTs ++, CAA	++ (Dementia)	-
**O’Riordan et al.** [25] (2002)	48	E280G (exon 8)	Cerebral minor Atrophy, CWP ++, CAA ++, Ischemic leukoencephalopathy	-	MI +, CD +
**Brooks et al**. [3] (2003)	48	Δ9 (G→A splice acceptor mutation)	-	+	MI +, CD ++
**Assini et al**. [26] (2003)	45	R278K (exon 8)	-	++ (Dementia)	MI +, CD +
**Hattori et al**. [27] (2004)	37	Missense mutation: Y154N	Temporal and parietal lobes Atrophy +	+	MI +, CD +
**Raman et al**. [28] (2007)	39	c.834A>C (Arg278Ser) (exon 8)	Cerebral and cerebellar Atrophy ++	+	MI ++
**Rudenskaya et al**. [29] (2007)	21–29	Thr421Ala	-	+	-
**Ringman et al.** [14] (2019)	24	F388S	Brainstem Atrophy	+	MI ++, CD
**Chelban et al**. [8] (2021)	30	Exon 9 heterozygous variant c.871A > C (Thr291Pro)	CWP ++, CAA	+	MI +
**Our case report (2025)**	54	c.845T>G p.(Leu282Arg)	CAA	-	CD ++
Cases without genetic confirmation **					
**Sodeyama et al.** [30] (1995)	53–57	-	Frontotemporal Atrophy ++, NFTs, SP	+	CD ++
**Crook, et al.** [19] (1998)	60	-	CWP, CAA, DCT ++, NFTs	++ (Progressive dementia)	MI +
**Houlden et al.** [11] (2000)	26–38	-	CWP	-	CD ++

CWPs: Cotton wool plaques. CAA: Cerebellar Amyloid Angiopathy. DCT: Degeneration of Corticospinal Tracts. NFTs: Neurofibrillary tangles. SP: Senile Plaques. MI: Motor impairment. CD: Cognitive deficits. Mutations in these pedigrees were not detected, but cases were presented as related to PSEN1 mutations. Criteria for introducing it in the table despite no mutation found is to keep a record of its clinical characteristics, since the absence of detected mutation is due to the original date of report in most cases. ** cases without confirmed mutations are included to document their clinical and neuropathological features. In most instances, the absence of genetic confirmation reflects the historical period of reporting, when technical or testing resources were unavailable. + mild-moderate, ++ severe. +++ severe-very severe.

## Figures and Tables

**Table 1:** Laboratory and genetic testing.

Laboratory Testing			
Biochemistry		Microbiological	
Glucose (mg/dL)	125	HIV	negative
Urea (mg/dL)	33	Hepatitis B	negative
Creatinine (mg/dL)	0.49	Hepatitis C	negative
Sodium (mE/L)	138	Brucella	negative
Potassium (mEq/L)	4.4	Mycoplasma pneumoniae	negative
Uric acid (mg/dL)	4.6	Treponema pallidum	negative
ALT (U/L)	23	Borrelia burgdorferi	negative
AST (U/L)	18	Herpes simplex virus	IgG positive
GGT (U/L)	30	Cytomegalovirus	IgG positive
ALP (U/L)	67	Epstein–Barr virus	IgG positive
Bilirubin (mg/dL)	0.8	Varicela zoster virus	IgG positive
LDH (U/L)	146	Measles	IgG positive
Cholesterol (mg/dL)	146		
Total proteins (g/dL)	6.2	**Immunology**	
Albumin (g/dL)	4.2	ANAs	negative
Calcium (mg/dL)	9.6	Rheumatoid factor (UI/mL)	7.01
Folic acid (ng/mL)	8.5	anti-tyroglobulin Abs	negative
Vitamin B12 (pg/mL)	267	anti-thyroid peroxidase Abs	negative
ACE (U/L)	18.9	anti-cardiolipin (IgM)	negative
TSH (mU/L)	1.32	anti-cardiolipin (IgG)	negative
25-OH Vit D (ng/mL)	18	anti-beta-2 glycoprotein (IgM)	negative
		anti-beta-2 glycoprotein (IgG)	negative
**Hemogram**		anti-aquaporin 4 Abs	negative
Leukocyte (×10^3^/uL)	7.5	anti-MOG Abs	negative
Neutrophils (×10^3^/uL)	5.1		
Lymphocytes (×10^3^/uL)	1.7	**Cerebrospinal fluid**	
Monocytes (×10^3^/uL)	0.5	Glucose (mg/dL)	74
Eosinophils (×10^3^/uL)	0.1	Proteins (mg/dL)	69
Basophils (×10^3^/uL)	0.1	Leukocytes (mm3)	5
Hemoglobin (g/dL)	15		
MCV (fL)	85.1	Oligoclonal bands	negative
Platelets (×10^3^/uL)	171		
**Biochemistry**		**Microbiological**	
Glucose (mg/dL)	125	HIV	negative
Urea (mg/dL)	33	Hepatitis B	negative
Creatinine (mg/dL)	0.49	Hepatitis C	negative
Sodium (mE/L)	138	Brucella	negative
Potassium (mEq/L)	4.4	Mycoplasma pneumoniae	negative
Uric acid (mg/dL)	4.6	Treponema pallidum	negative
ALT (U/L)	23	Borrelia burgdorferi	negative
AST (U/L)	18	Herpes simplex virus	IgG positive
GGT (U/L)	30	Cytomegalovirus	IgG positive
ALP (U/L)	67	Epstein–Barr virus	IgG positive
Bilirubin (mg/dL)	0.8	Varicela zoster virus	IgG positive
LDH (U/L)	146	Measles	IgG positive
Cholesterol (mg/dL)	146		
Total proteins (g/dL)	6.2	**Immunology**	
Albumin (g/dL)	4.2	ANAs	negative
Calcium (mg/dL)	9.6	Rheumatoid factor (UI/mL)	7.01
Folic acid (ng/mL)	8.5	anti-tyroglobulin Abs	negative
Vitamin B12 (pg/mL)	267	anti-thyroid peroxidase Abs	negative
ACE (U/L)	18.9	anti-cardiolipin (IgM)	negative
TSH (mU/L)	1.32	anti-cardiolipin (IgG)	negative
25-OH Vit D (ng/mL)	18	anti-beta-2 glycoprotein (IgM)	negative
		anti-beta-2 glycoprotein (IgG)	negative
**Hemogram**		anti-aquaporin 4 Abs	negative
Leukocyte (×10^3^/uL)	7.5	anti-MOG Abs	negative
Neutrophils (×10^3^/uL)	5.1		
Lymphocytes (×10^3^/uL)	1.7	**Cerebrospinal fluid**	
Monocytes (×10^3^/uL)	0.5	Glucose (mg/dL)	74
Eosinophils (×10^3^/uL)	0.1	Proteins (mg/dL)	69
Basophils (×10^3^/uL)	0.1	Leukocytes (mm^3^)	5
Hemoglobin (g/dL)	15		
MCV (fL)	85.1	Oligoclonal bands	negative
Platelets (×10^3^/uL)	171		
**Genetic testing**			

C12orf65, C19orf12, CA8, CACNA1A, CAPN1, CCDC88C, CCT5, CHP1, CLCN2, COASY, COL4A1, COL4A2, COX6B1, CPT1C, CSF1R, CSNK1D, CTNNB1, CYP27A1, CYP2U1, CYP7B1, DARS1, DARS2, DDHD1, DDHD2, DNA2, DSTYK, EARS2, ECHS1, EIF2B1, EIF2B2, EIF2B3, EIF2B4, EIF2B5, ELOVL4, ELP2, ENTPD1, ERLIN1, ERLIN2, EXOSC3, EXOSC8, FA2H, FAM126A, FARS2, FXN, GAD1, GALC, GAN, GBA2, GFAP, GJC2, GLB1, GLRX5, GM2A, GPT2, GRID2, GSX2, HEXA, HSD17B4, HSPD1, HTRA1, IBA57, IFIH1, IFRD1, KCNA1, KCNA2, KCND3, KCNK18, KIDINS220, KIF1A, KIF1C, KIF5A, KLC2, L1CAM, L2HGDH, LMNB1, LRP4, LYST, MAG, MARS2, MCOLN1, MECP2, MMADHC, MTPAP, NADK2, NALCN, NIPA1, NKX6-2, NOTCH3, NPC1, NPC2, NT5C2, OPA1, OPA3, OPHN1, PANK2, PCDH12, PDHX, PEX16, PGAP1, PLA2G6, PLP1, PNKD, PNPLA6, POLR1C, POLR3A, POLR3B, PRNP, PRRT2, PSAP, PSEN1, REEP1, REEP2, RNASEH2B, RTN2, SACS, SCN1A, SCN2A, SCN8A, SCP2, SDHA, SETX, SIL1, SLC16A2, SLC17A5, SLC1A3, SLC1A4, SLC25A15, SLC2A1, SLC33A1, SLC4A4, SOD1, SOX10, SPART, SPAST, SPG11, SPG21, SPG7, SPR, SPTAN1, STUB1, STXBP1, SYNE1, SYNJ1, TANGO2, TBCD, TECPR2, TFG, TPK1, TREM2, TREX1, TSEN54, TTC19, TTPA, TTR, TUBB4A, UCHL1, USP8, VAMP1, VLDLR, VPS11, VPS37A, VWA3B, WARS2, WASHC5, WDR45B, WDR48, WDR81, ZFR, ZFYVE26, ZFYVE27.

## Data Availability

Clinical data may be shared upon reasonable request to the corresponding author.

## Abbreviations

The following abbreviations are used in this manuscript:

EOAD	Early-onset Alzheimer’s Disease
AD	Alzheimer’s disease
PSEN1	Presenilin-1
CAA	Cerebral amyloid angiopathy
SP	Spastic paraparesis
CWP	Cotton wool plaques
ADAD	Autosomal-dominant Alzheimer’s Disease
